# Multitumor Case Series of Germline *BRCA1*, *BRCA2* and *CHEK2*-Mutated Patients Responding Favorably on Immune Checkpoint Inhibitors

**DOI:** 10.3390/curroncol28050280

**Published:** 2021-08-24

**Authors:** Lisa Kinget, Oliver Bechter, Kevin Punie, Philip R. Debruyne, Hilde Brems, Paul Clement, Eduard Roussel, Yannick Van Herck, Maarten Albersen, Marcella Baldewijns, Patrick Schöffski, Benoit Beuselinck

**Affiliations:** 1 Department of General Medical Oncology, Leuven Cancer Institute, University Hospitals Leuven, 3000 Leuven, Belgium; lisa.kinget@uzleuven.be (L.K.); oliver.bechter@uzleuven.be (O.B.); kevin.punie@uzleuven.be (K.P.); paul.clement@uzleuven.be (P.C.); yannick.vanherck@uzleuven.be (Y.V.H.); patrick.schoffski@uzleuven.be (P.S.); 2 Department of General Medical Oncology, AZ Groeninge, 8500 Kortrijk, Belgium; Philip.Debruyne@azgroeninge.be; 3 Medical Technology Research Centre (MTRC), School of Life Sciences, Faculty of Science and Engineering, Anglia Ruskin University, Cambridge CB1 1PT, UK; 4 Department of Human Genetics, University of Leuven, 3000 Leuven, Belgium; Hilde.brems@uzleuven.be; 5 Department of Urology, University Hospitals Leuven, 3000 Leuven, Belgium; eduard.roussel@uzleuven.be (E.R.); maarten.albersen@uzleuven.be (M.A.); 6 Department of Pathology, University Hospitals Leuven, 3000 Leuven, Belgium; marcella.baldewijns@uzleuven.be

**Keywords:** DNA damage response, immune checkpoint inhibitors, *BRCA1*, *BRCA2*, *CHEK2*

## Abstract

In recent years, immune checkpoint inhibitors (ICPI) have become widely used for multiple solid malignancies. Reliable predictive biomarkers for selection of patients who would benefit most are lacking. Several tumor types with somatic or germline alterations in genes involved in the DNA damage response (DDR) pathway harbor a higher tumor mutational burden, possibly associated with an increased tumoral neoantigen load. These neoantigens are thought to lead to stronger immune activation and enhanced response to ICPIs. We present a series of seven patients with different malignancies with germline disease-associated variants in DDR genes (*BRCA1*, *BRCA2*, *CHEK2*) responding favorably to ICPIs.

## 1. Introduction

Since the approval of the cytotoxic T-lymphocyte associated antigen-4 (CTLA-4) inhibitor ipilimumab for the treatment of metastatic melanoma in 2011, immune checkpoint inhibitors (ICPI) have become a cornerstone in the treatment of solid malignancies [1]. These monoclonal antibodies target immune checkpoint molecules such as CTLA-4, programmed death (PD)-1 or programmed death ligand (PD-L)1, through which they counteract the inhibition of T cells and promote the antitumor immune response. They are able to induce profound and durable responses in a subset of patients; however, selecting these patients remains a challenge that has given rise to substantial research for predictive biomarkers.

Tumor mutational burden (TMB) is a potential predictive biomarker that has shown promising results in malignant melanoma (MM) [2] and non-small cell lung carcinoma [3,4]. A higher TMB leads to a higher load of neoantigens that are recognized by cytotoxic T-lymphocytes, inducing an exhausted antitumoral immune reaction [5,6]. Upon treatment with ICPIs, the immune response is activated, leading to the antitumor immune response [7,8,9,10]. Previous pan-cancer research has demonstrated a response rate of 58% in patients with high TMB (>20 mut/Mb), whereas in patients with intermediate to low TMB the response rate is still around 20% [11].

Tumors with impaired DNA damage response (DDR) pathways, through bi-allelic mutations or copy number changes, usually harbor a higher TMB [12,13], leading to increased tumoral neoantigen load [14]. Additionally, increasing evidence has emerged of a direct link between DDR pathways and the innate immune system which can enhance the antitumor immune response independent of neoantigen burden [15,16]. Tumors arising in patients with germline disease-associated DDR variants could, therefore, display more favorable responses to ICPIs. Of the main pathways of DNA repair mechanisms (base excision repair, nucleotide excision repair, mismatch repair (MMR), homologous recombination (including Fanconi anemia) and nonhomologous end joining), remarkable responses to ICPI have been extensively demonstrated in MMR deficient tumors [17,18]. These groundbreaking trials led to FDA approval for the PD-1 antibody pembrolizumab for MMR deficient solid tumors regardless of primary tumor site [19].

In this study, we present a multitumor case series of patients carrying a germline DDR alteration displaying favorable outcomes on ICPIs.

## 2. Materials and Methods

### 2.1. Patient Selection

We retrospectively included patients treated with ICPIs who were screened for germline DNA alterations across two institutions (University Hospitals Leuven, Leuven, Belgium; AZ Groeninge Hospital, Kortrijk, Belgium). The patients were divided into two groups: with and without germline DDR alterations. As the predictive effect of MMR impairment on ICPIs has been well demonstrated [17,18], we did not include germline MMR alterations in the current study.

Tumor type was not a selection criterion, as far as there was an approved indication for ICPIs in monotherapy. Patients treated with ICPIs in combination with chemotherapy or targeted therapies were not included. Patients were treated according to institutional standards, following good clinical practice, with the PD-1-antibody nivolumab or pembrolizumab, the PD-L1-antibody atezolizumab, or the combination of an PD1-antibody with the CTLA-4-antibody ipilimumab.

We extracted baseline clinical characteristics from the patient files, including age, sex, tumor type, presence of other tumors, number of metastatic disease sites at the start of ICPI treatment, Eastern Cooperative Oncology Group Performance Status (ECOG PS), type of ICPI, best response, progression-free survival (PFS) and overall survival (OS). Response evaluation was based on Immune-related Response Evaluation Criteria In Solid Tumors (irRECIST) [20]. We extracted biochemical data with known prognostic impact at the start of ICPI treatment, including C-reactive protein (CRP), neutrophil/lymphocyte ratio (NLR) [21,22,23], albumin and lactate dehydrogenase (LDH). 

### 2.2. Genetic Testing

DNA testing was performed as part of clinical routine (A) to detect familial cancer syndromes in patients with multiple malignancies and/or young age at diagnosis, or (B) in patients with a family member carrying a known disease-associated germline variant. DNA was extracted from peripheral white blood cells by magnetic separation on a Chemagic 360 instrument (Perkin Elmer, Waltham, MA, USA) using the Chemagic DNA 4 k blood kit (Perkin Elmer, Waltham, MA, USA) according to the manufacturer’s instructions. Patients with a family member carrying a known disease-associated germline variant were tested specifically for that variant with Sanger sequencing. Patients without a known familial variant were tested with next generation sequencing (NGS) gene panels for hereditary cancer syndromes, with the panel composition expanded over the years as more genes for family cancer syndromes became known (time range 2016–2020). Initially, the BRCA hereditary cancer MASTR plus kit (Multiplicom/Agilent, Santa Clara, CA, USA) was used to detect small variants. Duplications and/or (multi) exonic deletions were investigated via MLPA (MRC Holland, Amsterdam, The Netherlands). From 2019 onwards, the HaloPlex panel (Agilent, custom design v2) was used to investigate disease-associated small variants and copy number variants. One patient was tested at another clinical center, where FamCanc panel and additional genetic testing for Precision-2 trial was performed. All panels included *BRCA1*, *BRCA2*, *PALB2*, *CHEK2* and *TP53*. The following reference sequences were used for the detected disease-associated variants: LRG_292t1 (*BRCA1*), LRG_293t1 (*BRCA2*), NM_007194.4 (*CHEK2*). The American College of Medical Genetics and Genomics guidelines were used to classify the variants [24]. In general, only class 4 (i.e., likely pathogenic) and class 5 (i.e., pathogenic) variants were reported.

### 2.3. Statistical Analysis

The main objective was to report cases of patients carrying DDR germline alterations and their favorable outcome on treatment with ICPIs. This study was not conceived for a formal comparison between subgroups. PFS and OS were estimated with Kaplan Meier survival analysis and compared with the log-rank test. Best responses were compared using Pearson’s Chi Squared test, and objective response rate (ORR) was analyzed using Fisher’s Exact test. Analyses were performed using R (version 4.0.03) (R Core Team, Vienna, Austria) software.

### 2.4. Ethical Approval

The study was approved by the Ethics Committee Research UZ/KULeuven (registration number S53479/S63833).

## 3. Results

### 3.1. Included Patients

We collected seven patient cases with germline DDR alteration carriers (*BRCA1* (*n* = 3), *BRCA2* (*n* = 3) and *CHEK2* (*n* = 1)). These patients were treated for MM, transitional cell carcinoma (TCC), renal cell carcinoma (RCC) or squamous cell carcinoma (SCC), with nivolumab (*n* = 3), pembrolizumab (*n* = 2), atezolizumab (*n* = 1) or ipilimumab/nivolumab (*n* = 1). We collected 13 patient cases for whom no class 4 or 5 variants were found. These patients were diagnosed with MM or RCC and treated with nivolumab (*n* = 8), pembrolizumab (*n* = 1) or ipilimumab/nivolumab (*n* = 4). Full patient details are reported in Table S1. Baseline prognostic parameters are reported in Table 1. Baseline albumin levels and ECOG PS were similar for both subgroups. The mean number of metastatic sites, LDH and NLR were higher in patients with germline alteration, but baseline CRP levels were higher in patients without. No statistical comparison was done due to small patient numbers.

### 3.2. Genetic Testing and Outcome on ICPI Treatment

**Figure 1:**
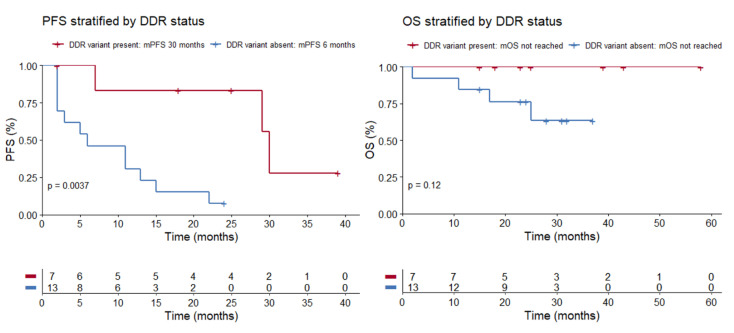
mPFS was 30 months in the patient group where a germline DDR variant was present, compared to 6 months in the patient group where a germline variant was absent (*p* = 0.0037, log-rank test). mOS was not reached, both in the patient group where a germline DDR variant was present and where it was absent (*p* = 0.12, log-rank test). Abbreviations: PFS = progression free survival, DDR = DNA damage response, mPFS = median progression free survival, OS = overall survival, mOS = median overall survival.

**Figure 2:**
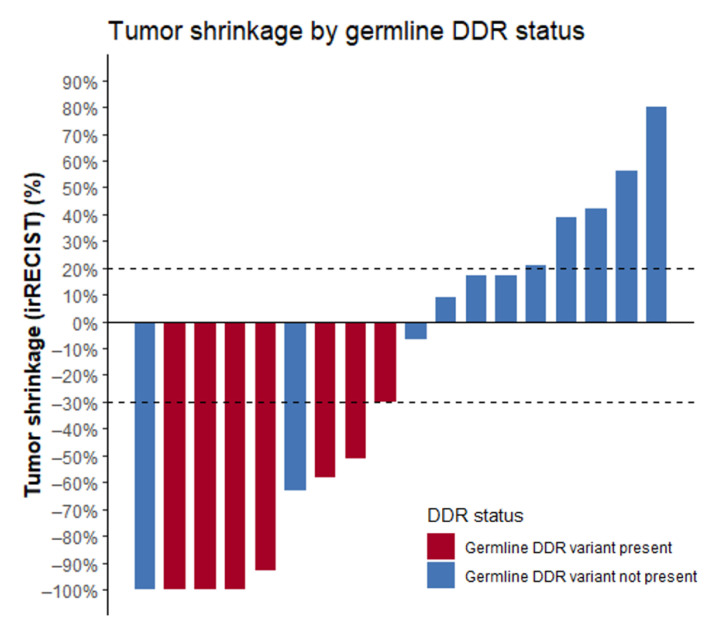
Waterfall plot displaying the best response to ICPIs according to irRECIST criteria. Note: two patients without DDR alterations were not included in this figure as precise tumor shrinkage was not available. Best response was defined as “clinical benefit”. Abbreviations: ICPIs = immune checkpoint inhibitors, DDR = DNA damage response.

The seven patients with detected germline alterations displayed very favorable outcomes on ICPIs. ORR was 86%, median PFS (mPFS) was 30 months (range 2 to 39 months), and OS was not reached (Figure 1; Table 2). At the time of analysis, response was ongoing in three patients and progression reached in three patients. One patient switched to second-line therapy because of severe toxicity on ICPI. Complete response (CR) was achieved in three patients. Best response on ICPI according to irRECIST is shown in Figure 2.

The 13 patients without detected alterations displayed modest outcomes. mPFS was rather short (6 months; range 2 to 39 months) and ORR low (15.4%), with one patient achieving a CR. At time of analysis, response was ongoing in one patient and progression reached in 12 patients. Median OS (mOS) was not reached. Best response on ICPI according to irRECIST is shown in Figure 2. The ORRs in these patients are in line with the expected outcomes in nonselected RCC and MM patients treated with nivolumab or ipilimumab/nivolumab.

Table 3 summarizes results of the genetic testing in each included patient. In six out of seven patients with a germline alteration, only the known familial disease-associated variant was tested without further germline testing. In one of these seven patients, germline testing for *BRCA1* was performed after somatic NGS revealed a *BRCA1* mutation in 50% of the alleles. In the 13 patients without germline alteration, several gene panels for hereditary cancer syndromes were used, all including *BRCA1*, *BRCA2*, *CHEK2*, Partner And Localizer Of BRCA2 *(PALB2)* and Tumor Protein P53 *(TP53)*.

## 4. Case Descriptions

### 4.1. Patient 1: BRCA1 (c.212+3A>G, p.?): Renal Cell Carcinoma

At the age of 65, this patient underwent radical prostatectomy for a localized prostate carcinoma (PC). Three years later, he underwent a nephrectomy for localized clear cell RCC. At the age of 70, he was diagnosed with localized rectal adenocarcinoma, treated with neoadjuvant radiotherapy, surgery and adjuvant chemotherapy. At the age of 81, local relapse with metastatic spread of the RCC was diagnosed with several liver lesions, confirmed by biopsy. Upon progression on sunitinib and axitinib, third-line treatment with nivolumab was initiated, leading to confirmed deep partial response (PR) (−93% irRECIST). Nivolumab was paused after 22 months because of sustained response but was reinitiated after 13 months due to progressive disease (PD). Upon further disease progression after 6 months, talazoparib was started in compassionate use. He currently has stable disease. OS was censored at 43 months. The corresponding phase 3 trial comparing nivolumab with everolimus in pretreated advanced RCC (Checkmate 025) demonstrated a mPFS of 4.6 months, an ORR of 25% and a mOS of 25 months in nivolumab-treated patients [25]. This case was previously published by Beulque et al. [26].

### 4.2. Patient 2: BRCA2 (c.6644_6647del, p.Ty2215Serfs*13): Squamous Cell Carcinoma of Unknown Origin

At the age of 68, this patient underwent a radical prostatectomy for a localized PC. Subsequently, a local relapse was treated with radiotherapy and a biochemical recurrence by androgen deprivation therapy. Three years later, he presented with a symptomatic brain metastasis. After complete resection, pathologic evaluation showed it to be a SCC metastasis. Additional staging demonstrated diffuse lymph node and bone metastases, with a low prostate specific antigen (PSA) level. First-line treatment with cisplatin-fluorouracil proved inefficient. Nivolumab in second line led to a PR (−58% irRECIST), which is ongoing after 25 months. Meanwhile, docetaxel was associated because of rapidly rising PSA levels, leading to a biochemical response and discontinuation of docetaxel after 7 months. This patient was censored for OS at 25 months. In a corresponding phase 3 trial in patients with head and neck SCC with recurrent disease on platinum-based chemotherapy (CheckMate 141), nivolumab-treated patients had a mPFS of 2 months, an ORR of 13.3% and a mOS of 7.5 months [27].

### 4.3. Patient 3: CHEK2 (c. 1100del, p.Thr367Metfs*15): Malignant Melanoma

Patient 3 was diagnosed with a localized *BRAF*-wild type MM at the age of 31 and treated with wide excision. One year later, staging showed lung, lymph node, liver and spleen metastases. First-line systemic therapy with pembrolizumab was initiated. After two months, staging showed a slight increase of liver and lymph node metastases but complete response (CR) of lung and spleen metastases. Surgical resection of the liver metastasis and axillary lymph nodes showed pathological confirmed CR. Pembrolizumab is still ongoing after 39 months with sustained CR. In the KEYNOTE-006 trial investigating pembrolizumab vs. ipilimumab in advanced melanoma, pembrolizumab-treated patients had a mPFS of 4.1 months and an ORR of 32.9% [28]. 

### 4.4. Patient 4: BRCA2 (c.516+1G>A, p.?): Malignant Melanoma

Patient 4 was diagnosed with a *BRAF*-mutated MM at the age of 57, with lymph node, lung and brain metastases. Shortly after the start of ipilimumab/nivolumab in first-line, he developed severe auto-immune meningitis and pneumonitis. Staging showed a decrease of the largest brain metastasis and adenopathies (−29% irRECIST); however two subcentrimetric brain metastasis had slightly increased. Due to severe toxicity, ICPI was switched to dabrafenib-trametinib with an ongoing PR after 13 months of therapy. The patient was censored for PFS at two months and for OS at 23 months. The corresponding phase 3 trial in patients with previously untreated advanced melanoma (CheckMate-067) demonstrated a mPFS of 11.5 months, an ORR of 57.6% and a mOS of more than 60 months in the patient group treated with ipilimumab-nivolumab [29]. 

### 4.5. Patient 5: BRCA1 (c.5186T>A, p.Leu1729Gln): Malignant Melanoma

Patient 5 discovered a breast nodule at age 38, which was confirmed to be a *BRAF*-mutated MM metastasis with additional liver and lymph node metastases. First-line therapy with dabrafenib-trametinib led to a PR. Tumor tissue NGS showed a *BRCA1* class 3 variant in 55% of the reads. Subsequent testing confirmed it to be a germline variant. This patient had a second degree female relative diagnosed with breast cancer at the age of 48 (no germline testing available for this relative). Therefore, we included this patient in this study even though the variant was not of class 4 or 5. After 27 months, for multifocal progressive disease, ipilimumab/nivolumab was started, leading to CR. After 30 months, staging showed a new breast nodule with axillary lymph nodes. Pathological examination couldn’t differentiate between local MM relapse, clinically the more likely alternative, or triple negative breast cancer. However, on a multitumor board it was advised to consider this new tumor as breast carcinoma and to offer a potentially curative treatment. Immunotherapy was discontinued. After mastectomy and adjuvant chemo-radiotherapy, she has been without evidence of disease for 21 months. To avoid bias through a more favorable interpretation of results, we considered the new tumor in the breast as a MM relapse after 30 months of ICPI treatment for this study, and not as a new breast carcinoma. The patient was censored for OS at 58 months. As mentioned above, in the CheckMate-067 trial in advanced melanoma patients, ipilimumab-nivolumab treated patients had a mPFS of 11.5 months, an ORR of 57.6% and a mOS of more than 60 months [29]. 

### 4.6. Patient 6: BRCA2 (c.4936_4939del, p.Glu1646Glnfs*23): Transitional Cell Carcinoma

At the age of 63, patient 6 was diagnosed with stage IV TCC. First-line therapy with cisplatin-gemcitabine led to PR. At disease progression, he received the PARP-inhibitor olaparib. This treatment was discontinued after two months because of PD. Atezolizumab was initiated and is currently, after 10 months, ongoing with a PR (−51% irRECIST). At 7 months of therapy, his disease progressed. He was censored for OS at 15 months. In the IMvigor 211 trial comparing atezolizumab with chemotherapy in platinum-pretreated patients, patients in the ICPI arm had a mPFS of 2 months, an ORR of 13.4% and a mOS of 8.6 months [30,31].

### 4.7. Patient 7: BRCA1 (c.212+3A>G, p.?): Transitional Cell Carcinoma

At the age of 57, patient 7 underwent neoadjuvant chemotherapy and radical cystoprostatectomy for a muscle invasive TCC. Several months later pembrolizumab was initiated for diffuse metastatic spread (PD-L1 combined positive score > 10), leading to a CR. Therapy is currently ongoing, 18 months after start. In the corresponding phase 3 trial, previously untreated patients with advanced urothelial carcinoma who were treated with pembrolizumab in monotherapy had an ORR of 30.3% and a mOS 15.6 months (KEYNOTE-361) [32].

## 5. Discussion

We report on 7 patients with a germline *BRCA1*, *BRCA2* or *CHEK2* alteration with distinct metastatic malignancies displaying favorable responses on ICPIs in terms of RR, number of CRs and mPFS. In a group of 13 patients for whom genetic testing did not show evidence of germline alterations, outcomes were more modest. Additionally, the outcomes of patients with DDR alterations are generally more favorable than outcomes demonstrated in the phase 3 trials investigating the respective ICPI agent in each cancer type. 

Our findings further support the concept that pre-existing germline alterations in DNA repair systems could enhance response to ICPIs. Impaired DDR pathways lead to higher levels of intratumor genomic instability, more potential for neoantigens and higher immunogenicity [3,8]. Additionally, accumulating damaged DNA fragments in the cytosol of cells with impaired DDR pathways activate the type I interferon response [33]. The resulting stimulation of the innate antitumor immune response is correlated with durable responses to ICPIs [34]. Of the main pathways of DNA repair mechanisms (base excision repair, nucleotide excision repair, mismatch repair, homologous recombination (including Fanconi anemia) and nonhomologous end joining), remarkable responses to ICPI have been demonstrated in MMR deficient tumors [17,18]. In 2017, pembrolizumab received FDA approval for MMR deficient solid tumors, regardless of primary tumor site [19]. Furthermore, pembrolizumab recently received FDA approval for tumors with high TMB (≥10 mut/Mb) [35]. A noncomprehensive list of reports showing similar findings in other DDR pathways is shown in Table 4. Evidence appears strongest and most concordant in bladder carcinoma, followed by RCC, and is more conflicting in prostate carcinoma and melanoma. However, most publications report a positive correlation between the presence of somatic DDR alterations and clinical outcome in a variety of different cancers. Despite the consistency of this association, one needs to take into account that the level of evidence is fairly low, since most publications encompass case reports or small case series. 

Limitations of this study are the retrospective nature of the data and small patient number, subject to recall bias. Relapses were not routinely confirmed through biopsy. Molecular data about TMB or somatic DDR alterations were not available and therefore extrapolated from germline data. However, it is unlikely that this is a source of inconsistency. 

## 6. Conclusions

Taken together, our data further support the existing evidence for a potential role of germline DDR disease-associated variants as predictive biomarkers for ICPI response, which is worthwhile to be further studied prospectively in several tumor types, particularly in bladder carcinoma and RCC. Our data also suggest that patients with metastatic cancers harboring germline DDR mutations should be offered ICPIs, and that larger randomized clinical trials comparing standard of care with ICPI in first line should be conducted in this rare population of cancer patients.

## Figures and Tables

**Table 1:** Baseline characteristics.

	Germline DDR Variant Present (*n* = 7)	Germline DDR Variant Absent (*n* = 13)
**Age at diagnosis (median, range)**	59 (31–73)	57 (43–71)
**Gender**	5 males—2 females	7 males—6 females
**Tumor Type (*n*)**		
**Malignant melanoma**	3	2
**Squamous cell carcinoma of unknown origin**	1	0
**Renal cell carcinoma**	1	11
**Transitional cell carcinoma**	2	0
**Second Tumor (*n*)**		
**Prostate adenocarcinoma**	2	2
**Colorectal adenocarcinoma**	1	2
**Transitional cell carcinoma**	0	1
**Breast cancer**	0	5
**Malignant melanoma**	0	2
**Basocellular carcinoma (skin)**	0	2
**Squamous cell carcinoma (skin)**	0	1
**Endometrial cancer**	0	1
**Clinical and Biochemical Characteristics**		
**Number of metastatic sites (mean, SD)**	3.57 ± 1.8	2.23 ± 1.0
**CRP (mg/L) (mean, SD)**	28.5 ± 63.0	51.2 ± 77.9
**Albumin (g/L) (mean, SD)**	40.9 ± 5.34	39.3 ± 3.9
**NLR (mean, SD)**	5.85 ± 7.1	3.54 ± 2.6
**LDH (U/l) (mean, SD)**	237.9 ± 87.2	207.2 ± 65.2
**ECOG Performance Status**		
**ECOG 0 (*n*, %*)***	5 (71.4%)	8 (61.5%)
**ECOG 1 (*n*, %*)***	2 (28.6%)	5 (38.5%)
**Type of ICPI**		
**Nivolumab**	3 (42.9%)	8 (61.5%)
**Ipilimumab and nivolumab**	1 (14.3%)	4 (30.8%)
**Pembrolizumab**	2 (28.6%)	1 (7.7%)
**Atezolizumab**	1 (14.3%)	0 (0%)

Abbreviations: CRP = C-reactive protein; SD = standard deviation; NLR = neutrophil to lymphocyte ratio; LDH = lactate dehydrogenase; ECOG PS = Eastern Cooperative Oncology Group performance status; ICPI = immune checkpoint inhibitor.

**Table 2:** Best response according to irRECIST in the patient groups with or without germline DDR variant.

Germline DDR Status	Partial Response	Stable Disease	Progressive Disease	Clinical Benefit	*p*-Value (Chi-Square Test)
Germline DDR variant present	6/7 (86%)	1/7 (14%)	0/7 (0%)	0/7 (0%)	0.02
Germline DDR variant absent	2/13 (15.4%)	4/13 (30.8%)	5/13 (38.5%)	2/13 (15.4%)	

Abbreviations: DDR = DNA damage response.

**Table 3:** Genetic testing performed in each patient.

**Patient**	**Genetic Variant Identified**	**Genetic Screening Performed**
1	*BRCA1* (c.212+3A>G, p.?)	Analysis of known family variant
2	*BRCA2* (c.6644_6647del, p.Ty2215Serfs*13)	Analysis of known family variant
3	*CHEK2* (c.1100del, p.Thr367Metfs*15)	Analysis of known family variant
4	*BRCA2* (c.516+1G>A, p.?)	Analysis of known family variant
5	*BRCA1* (c.5186T>A, p.Leu1729Gln)	*ATM*, *BRIP1*, *CDH1*, *CHEK2*, *MLH1*, *MSH2*, *MSH6*, *NBN* (only c.657_661del), *PALB2*, *PTEN, RAD51C, RAD51D, TP53* Additional testing: NGS sequencing of tumor tissue
6	*BRCA2* (c.4936_4939del, p.Glu1646Glnfs*23)	Analysis of known family variant
7	*BRCA1* (c.212+3A>G, p.?)	Analysis of known family variant
8	No class 4 or 5 variant found	HaloPlex panel
9	No class 4 or 5 variant found	HaloPlex panel
10	No class 4 or 5 variant found	HaloPlex panel
11	No class 4 or 5 variant found	HaloPlex panel
12	No class 4 or 5 variant found	HaloPlex panel
13	No class 4 or 5 variant found	HaloPlex panel
14	No class 4 or 5 variant found	HaloPlex panel
15	No class 4 or 5 variant found	HaloPlex panel
16	No class 4 or 5 variant found	BRCA hereditary cancer MASTR plus kit
17	No class 4 or 5 variant found	BRCA hereditary cancer MASTR plus kit
18	No class 4 or 5 variant found	BRCA hereditary cancer MASTR plus kit
19	No class 4 or 5 variant found	FamCanc + Precision 2 trial (BMSO)
20	No class 4 or 5 variant found	HaloPlex panel

Abbreviations: *ATM* = Ataxia Telangiectasia Mutated; *BRCA1* = *BRCA1* DNA Repair Associated; *BRCA2* = *BRCA2* DNA Repair Associated; *BRIP1* = *BRCA1* interacting protein C-terminal helicase 1; *CDH1* = cadherin 1; *CHEK2* = checkpoint kinase 2; *MLH1* = MutL Homolog 1; *MSH2* = MutS Homolog 2; *MSH6* = MutS Homolog 6; *NBN* = Nibrin; *PALB2* = Partner And Localizer Of *BRCA2*; *PTEN* = Phosphatase And Tensin Homolog; *RAD51C* = RAD51 paralog C; *RAD51D* = RAD51 paralog D; *TP53* = Tumor Protein P53; NGS = next generation sequencing.

**Table 4:** Literature evidence regarding the effect of DDR disease-associated variants on the response to ICPIs.

Authors	*n*	Somatic or Germline	ICPI	Concordant with DDR Hypothesis	Findings
				**Pan-cancer**	
Zhou et al. [36]	141	Somatic	ICPI in monotherapy or combination	Yes	Patients with somatic *BRCA2* alterations had improved OS (median OS 31 vs. 18 months, *p* = 0.02). Patients with *BRCA2* altered tumors with low TMB had comparable OS with patients with high TMB tumors (median OS 44 vs. 41 months; *p* < 0.001).
**Metastatic urothelial carcinoma**					
Teo et al. [37]	60	Somatic	Anti-PD(L)1 antibodies in monotherapy	Yes	Response rate of 80% in patients with deleterious DDR alteration (*n* = 18), 54% in patients with DDR VUS (*n* = 15) and 19% in wild type tumors (*n* = 17) (*p* < 0.001). Median PFS not reached, 15.8 and 2.9 months, respectively, and median OS not reached, 23.0 and 9.3 months, respectively.
Joshi et al. [38]	53	Somatic	Anti-PD(L)1 antibodies	Yes	DDR alterations, somatic or germline, were associated with trend towards longer OS. Increased number of DDR alterations were associated with trend for higher ORR.
Powles et al. [39]	559	Somatic	Avelumab versus BSC	Yes	DDR alterations were associated with improved OS when treated with ICPI (HR 0.65; 95%CI 0.504–0.847) compared to BSC. Association was not observed in DDR wild type tumors (HR 0.89; 95%CI 0.489–1.612).
**Metastatic renal cell carcinoma**					
Labriola et al. [40]	34	Somatic	Nivolumab, ipilimumab—nivolumab or pembrolizumab	Yes	68.8% of patients with disease control (*n* = 16) had enrichment in somatic DDR alterations vs. 38.9% of patients with PD (*n* = 18) (*p* = 0.03).
Ged et al. [41]	107	Germline (27%) Somatic (73%)	Anti-PD1 monotherapy (68%) and combination ICPIs (32%)	Yes	19 patients had deleterious DDR alterations and 88 patients wild type/VUS DDR. Deleterious DDR was associated with improved OS on ICPI (*p* = 0.049). This effect was not seen in control group of 118 patients treated with angiogenesis inhibitors.
**Metastatic malignant melanoma**					
Hugo et al. [42]	469	Somatic	Nivolumab and pembrolizumab	Yes	28 patients had somatic *BRCA2* mutation. *BRCA2* mutations were significantly more frequent in responders compared to nonresponders (OR 6.2, *p* = 0.002).
Amaral et al. [43]	4	Germline	Combination ICPIs	No	None of the patients (two with *BRCA2*, one with *BAP1* and one with *PALB2* germline alteration) responded well.
**Metastatic ovarian carcinoma**					
Matsuo et al. [44]	6	Germline	Nivolumab	Yes	In these six heavily pretreated patients with germline *BRCA1*/2 mutations, ORR was 67%.
Liu et al. [45]	134	Somatic of germline	ICPIs	No	31 patients had deleterious somatic or germline *BRCA1*/2 mutations. No association was found between mutation status and response to ICPIs.
**Metastatic castration resistant prostate carcinoma**					
Boudadi et al. [46]	15	Somatic	Ipilimumab—nivolumab	Yes	Six out of these 15 patients with an aggressive subtype of AR-V7 expressing prostate carcinoma carried a somatic DDR mutation (three *BRCA2*, two in *ATM* and one in *ERCC4*) and showed improved PFS (HR 0.31, *p* = 0.01) compared to the nine patients without DDR mutations.
Markowski et al. [47]	3	One germline, two somatic	PD-1 inhibitors	Yes	Three patients with inactivating *BRCA2* or *ATM* mutations showed profound and durable response to ICPI.
Antonarakis et al. [48]	153	Somatic	Pembrolizumab	No	29 patients with somatic DDR mutations, response to ICPI was not associated with mutational status.
**Case reports in other tumors**					
Pang et al. [49]	1	Germline	Pembrolizumab	Yes	Patient with metastatic PDAC with germline and somatic *PALB2* mutation, had a durable PR.
Boeck et al. [50]	1	Germline	Pembrolizumab	No	Patient with metastatic PDAC, with a germline *PALB2* mutation, had PD as best response.
Dizon et al. [51]	1	Germline and somatic	Pembrolizumab	Yes	Patient with high grade Mullerian adenocarcinoma, with germline *BRCA1* and somatic *BRCA2* mutation, had a CR.
Santin et al. [52]	1	Somatic	Nivolumab	Yes	Patient with hypermutated endometrial tumor with a *POLE* mutation had a profound PR.
Momen et al. [53]	1	Germline	Pembrolizumab	Yes	Patient with xeroderma pigmentosum (germline *XPC* mutation) and metastatic angiosarcoma (somatic *POLE* mutation and high TMB) achieved PR.
Johanns et al. [54]	1	Germline	Pembrolizumab	Yes	Patient with a hypermutated glioblastoma and germline *POLE* mutation showed evidence of clinical and immunological response to ICPI.

Note: for this literature overview, we did not consider studies on the impact of MMR because this has already been studied intensively and ICPI have been approved by the FDA in a tumor-agnostic way.

## Data Availability

The data presented in this study are available in Table S1.

## Supplementary Materials

The following are available online at https://www.mdpi.com/article/10.3390/curroncol28050280/s1, Table S1: Characteristics of included patients.

## Abbreviations

mRCC = metastatic renal cell carcinoma; ICPI = immune checkpoint inhibitor; OS = overall survival; TMB = tumor mutational burden; PD-1 = programmed death receptor 1; PD-L1 = programmed death receptor ligand 1; VUS = variant of unknown significance; DDR = DNA damage response; mUC = metastatic urothelial carcinoma; ORR = objective response rate; BSC = best supportive care; *ERCC4* = Excision Repair Cross-Complementation Group 4; *ATM* = Ataxia Telangiectasia Mutated; PDAC = pancreatic ductal adenocarcinoma; *PALB2* = Partner and Localizer of Breast Cancer 2; PR = partial response; PD = progressive disease; CR = complete remission; HRR = homologous recombinational repair; MSI = microsatellite instability; PFS = progression-free survival; mCRPC = metastatic castration resistant prostate cancer; *POLE* = DNA Polymerase Epsilon; *MSH6* = mutS homolog 6.
